# Extracellular Vesicles Function as Bioactive Molecular Transmitters in the Mammalian Oviduct: An Inspiration for Optimizing in Vitro Culture Systems and Improving Delivery of Exogenous Nucleic Acids during Preimplantation Embryonic Development

**DOI:** 10.3390/ijms21062189

**Published:** 2020-03-22

**Authors:** Bo Fu, Hong Ma, Di Liu

**Affiliations:** 1 Institute of Animal Husbandry Research, HeiLongJiang Academy of Agricultural Sciences, Harbin 150086, China; fubohao810@163.com (B.F.); mahong197400@163.com (H.M.); 2 Key Laboratory of Combine of Planting and Feeding, Ministry of Agriculture of the People’s Republic of China, Harbin 150086, China

**Keywords:** preimplantation embryo, oviduct, extracellular vesicles, oviductosomes, transgenic animal

## Abstract

Two technologies, in vitro culture and exogenous gene introduction, constitute cornerstones of producing transgenic animals. Although in vitro embryo production techniques can bypass the oviduct during early development, such embryos are inferior to their naturally produced counterparts. In addition, preimplantation embryos are resistant to the uptake of exogenous genetic material. These factors restrict the production of transgenic animals. The discovery of extracellular vesicles (EVs) was a milestone in the study of intercellular signal communication. EVs in the oviduct, known as oviductosomes (OVS), are versatile delivery tools during maternal–embryo communication. In this review, we discuss the important roles of OVS in these interactions and the feasibility of using them as tools for transferring exogenous nucleic acids during early development. We hypothesize that further accurate characterization of OVS cargoes and functions will open new horizons for research on maternal–embryo interactions and enhance the production of transgenic animals.

## 1. Introduction

The mammalian oviduct comprises the infundibulum, ampulla and isthmus. Given that after being transferred to the uterus, embryos derived from intracytoplasmic sperm injection or in vitro fertilization can establish a pregnancy and that normal oviduct–embryo communication has been bypassed, the role of the oviduct has been underestimated. However, it is unanimously accepted that the oviduct is not just a tract that connects the ovary to the uterus and serves simply for transporting the early embryos. In fact, it provides the best environment for the embryos before they enter the uterus [1]. Many kinds of reproductive events (e.g., gamete maturation, gamete transport, sperm capacitation, fertilization and early embryo development) take place in the oviduct. Inside this tubular structure—the oviductal microenvironment—is where the first maternal–embryo cross-talk initiates, as it contains key molecules involved in such communication. The quality of embryos produced in vitro is inferior to those developed in vivo, and the quality of embryos without coculture is also inferior to those embryos cocultured with oviduct epithelial cells; the deficiencies were manifested in terms of abnormal large offspring, lower pregnancy rates and aberrant gene expression profiles [2,3,4,5]. All these indicate that the conditions of in vitro embryo production are far from normal and that oviduct–embryo communication is important during preimplantation embryonic development.

Exosomes and microvesicles, collectively termed extracellular vesicles (EVs), are attracting increasing attention. EVs function as evolutionarily conserved bioactive transmitters of intercellular communication, and participate actively in cell-to-cell cross-talk [6,7]. Because proteins, messenger (m)RNAs and small RNAs are loaded by EVs, these lipid bilayer-enclosed membrane vesicles enable communication between cells [8,9]. EVs containing proteins, mRNAs and small RNAs have also been identified in oviductal fluid (known as oviductosomes or OVS), indicating that they might function as transmitters of bioactive molecules and mediate oviduct–embryo communication [10,11,12,13]. The study of OVS is providing us with new insights into communication between the oviductal epithelium and embryos.

Although the in vitro microenvironments for culturing preimplantation embryos are designed to be similar to the composition of oviductal fluid, the culture media currently available still lack EVs derived from oviductal fluid, which means that such systems bypass the normal interactions between oviducal epithelial cells and embryos. Investigating the mechanisms underlying such communication mediated by OVS might provide novel methods for reformulating or optimizing in vitro culture (IVC) media. In addition, preimplantation embryos are remarkably resistant to the uptake of exogenous substances such as drugs and genetic material. Nucleic acid delivery methods, such as microinjection, in vitro electroporation, viral transduction and liposomal transfection, have defects [14]. Given that EVs—natural nanosized shuttles—are novel tools for delivering biomolecules to target cells [15,16,17,18], exploring the characteristics of OVS might also provide novel methods for delivering exogenous nucleic acids into preimplantation embryos.

This review aims to outline the key roles played by OVS during communication between the oviduct and preimplantation embryos and the feasibility of using them as tools for transferring exogenous genetic material into such embryos. Direct supplementation of embryos with OVS should enhance their developmental capacity; delivery of exogenous nucleic acids into preimplantation embryos using OVS might also allow us to bypass invasive delivery procedures and develop novel transgenic delivery technologies.

## 2. General Aspects of EVs 

According to the Minimal Information for Studies of Extracellular Vesicles (MISEV) 2018 guidelines for EVs, the generic term “EVs” refers to particles naturally released from the cell that are delimited by a lipid bilayer and cannot replicate. Due to the difficulty of distinguishing EVs, these vesicles are classified into large (L)-EVs and small (S)-EVs based on their diameter [19]. The L-EVs, larger vesicles than 200 nm, include microvesicles/ectosomes, apoptotic bodies and large oncosomes, while S-EVs include exosomes and exomeres. Microvesicles (0.2–0.5 μm) are large extracellular vesicles originated from the plasma membrane by direct shedding in response to cellular activation or stress, apparently in a manner similar to that of retroviruses [20,21,22,23,24]. Baig et al. [25] also found that microvesicles originate at special lipid raft domains of the plasma membrane. When cells undergo apoptosis, they dissociate into membrane-bound apoptotic bodies (0.8–5 μm), which might contain genomic DNA [20,26]. In addition, in cancer cells, the larger EVs (1–10 μm), termed large oncosomes, can bleb off the cell membrane [20,27]. Exosomes (50–100 nm), a subclass of (S)-EVs, are produced via lyso-endosomal pathways. Exosomes, or intraluminal vesicles, are formed from the inward curve of endosomal membranes during the maturation of multivesicular endosomes (MVEs). If MVEs fuse with the plasma membrane, exosomes are released into the extracellular space [7]. Tetraspanins such as CD9, CD63 and CD81, which have been shown to bind to endosomal sorting complexes required for transport (ESCRT) machinery, are enriched in exosomes. The other (S)-EVs that are nonmembranous nanoparticles, known as exomeres, have also been identified through employing asymmetric flow field-flow fractionation [28]. Due to the overlapping characteristics of the major subclasses of EVs, it is difficult to distinguish among them. Thus, according to the Minimal Information for Studies of Extracellular Vesicles (MISEV) 2018 guidelines, unless authors can establish specific markers of subcellular origin, it is advisable to use operational terms for EVs, with a description of physical characteristics, such as size, biochemical composition, conditions, or cell of origin, to distinguish subsets of EVs [19,29].

Apoptotic bodies also have signaling functions, which carry “find-me” and “eat-me” molecular signals to attract phagocytes to apoptotic sites and promote apoptotic cell clearance [30,31]. Other EVs, such as exosomes and microvesicles, are constantly released by normal viable cells, while apoptotic bodies are released exclusively during apoptotic cell death [26]. In this review, we will focus on exosomes and microvesicles for the remainder of the discussion.

EVs contain a number of cargoes such as proteins, mRNAs and micro (mi)RNAs, which have been reviewed by Raposo et al. [32]. Besides these cargoes, it is now known that genomic DNA (gDNA) fragments in EVs are transportable between cells, then increase the gDNA-coding mRNA and protein expressions in the recipient cells, eventually affecting the function of recipient cells; for instance, the AT1 receptor DNA in EVs could increase AT1 receptor expression, then stimulate Na^+^-K^+^ ATPase activity in recipient HEK293 cells [33]. In addition, EVs derived from embryos also contain DNA fragments that represent the full murine genome, which may be useful for embryo chromosomal noninvasive diagnosis [34].

After first being described in sheep reticulocytes by Pan et al. [35], EVs have been identified in diverse extracellular fluids [36,37,38,39,40,41]. Previously, the process of releasing EVs had been considered as a means of discarding nonfunctional cellular components from the cell [42]. However, in 2007, Valadi et al. [43] demonstrated that exosomes derived from various cell types contain a large number of RNAs, including mRNAs, microRNAs, 18S or 28S ribosomal RNAs, and exosomes are transmitters of genetic material between cells. It is well known that EVs are not just waste carriers and that they act as transmitters for intercellular communication and signaling. They are considered to be evolutionarily conserved mediators of intercellular communication thanks to their capacity for exchanging nucleic acids, lipids and proteins between cells [44,45,46].

Capitalizing on the physicochemical and biochemical properties of EVs, many techniques, such as ultracentrifugation, ultrafiltration, immunoaffinity capture and polymer-based precipitation, have been developed for their isolation. Unfortunately, these isolation techniques also carry a unique set of advantages and disadvantages, as reviewed by Pin et al. [47]. Powered by the precise nanoscale liquid and particle control capacity of microfluidic-based devices, microfluidic platforms, which enable fast, low cost, portable and small volumes of liquid samples, might be ideal tools for isolating EVs. Based on size-based separation, immunoaffinity-based separation or dynamic separation, microfluidic separation methods provide many advantages (including higher recovery, higher purity, higher throughput, higher resolution and sensitivity, short separation time and real-time observation) in separating EVs. Although each microfluidic separation method has specific limitations, combining dynamic, size-based and immunoaffinity-based microfluidic methods into one integrated device—a streamlined strategy—might overcome the disadvantages of microfluidic methods, ultimately providing opportunities for on-chip isolation of exosomes [48,49,50].

## 3. Oviductosomes Participate in Communication between Oviduct and Embryos

The first stages of embryo development in eutherian mammals occur in the oviduct, where the embryo spends around 4–8 days, depending on species. Oviductal fluid contains factors that can enhance the quality of embryos [51,52]. The mechanism underlying the delivery of these bioactive factors into preimplantation embryos is fascinating. Release of EVs appears to be an evolutionarily conserved process. Thus, EVs should also appear in oviductal fluid. The term OVS is used to refer to both exosomes and microvesicles detected in the oviductal fluid. After OVS were identified for the first time in murine oviductal fluid [53], they have also been identified in the oviductal fluid of other species [54,55,56]. 

OVS are released from the oviductal epithelium, then internalized by preimplantation embryos, ultimately improving embryo development and quality through bioactive molecules contained in these vesicles. Increasing evidence also indicates that the oviduct can enhance embryo development and quality with the participation of OVS [10,11,12,57]. Generally, OVS mainly include proteins, mRNAs and miRNAs. Almiñana et al. [11] isolated OVS from bovine oviductal fluid. Besides the proteins characteristic of an exosomal proteomic signature, they also identified the presence of oviduct-specific glycoprotein precursor 1 (OVGP1). OVGP1 can bind to the zona pellucida (ZP) of the oocyte or early embryo, then interact with it and eventually cause the ZP to block polyspermic fertilization and ensure normal embryonic development [52,58]. It is also known that during ciliary beating and muscular contractions of the oviduct, OVGP1 can also prevent nutrients and ions from being dispersed, thereby stabilizing the microenvironment of early embryos [59]. mRNAs that act on embryos also have been identified in OVS. They are rich in mRNAs encoding ribosomal proteins, which might participate in protein translation, immune signaling and development [60]. In addition, the mRNAs associated with histone methyltransferases (e.g., EHMT1, EHMT2, EZH1, KMT2A, KMT2B, KMT2C, PHF1, PHF2, PRMT5, SETD1A, and SETD2), histone demethylases (e.g., ARID5B, JMJD1C, KDM2A, KDM3B, KDM5B, KDM5C, and KDM6B) and the DNA methyltransferase (DNMT1) have also been identified in OVS, indicating that chromatin modification and epigenetic regulation in the early embryo might be controlled by the oviduct via OVS [13]. Surprisingly, tumor protein translationally-controlled 1 (TPT1), which can induce the activation of pluripotency genes such as *Nanog* and *Oct4* in oocytes, has also been identified in OVS [61]. Micro (mi)RNAs could promote degradation or inhibit translation by binding to complementary sequences in target transcripts, eventually being responsible for fine-tuning many gene regulatory networks. Regulatory miRNAs in OVS, such as miR-375, might be associated with amino sugar and nucleotide sugar metabolisms and be involved in cell fate determination in early embryos [13,62]. In addition, by using an integrated platform linking miRNA targets and functions, a comprehensive analysis of miRNAs contained in OVS and their potential target genes in embryos suggested that many miRNA target genes are involved in embryo development, embryo morphology or implantation [13]. Several researchers have demonstrated that several key genes for embryo development (e.g., *Bcl2*, *Cdk6* and *c-Myc*), are also targets of OVS miRNAs [63,64,65]. Currently, Qu et al. [66] have shown that neurohormone contained in OVS also improved the developmental competence of embryos. Melatonin, which is involved in antioxidant activity, is abundantly present in OVS. The addition of these OVS into the embryo culture media led to a significant downregulation of reactive oxygen species (ROS) and 5-methylcytosine (5-mC) as well as an increase in the blastocyst rate of embryos, which can be inhibited by the addition of luzindole (a melatonin receptor agonist). Having these results in mind, we should be aware of the fact that the molecular cargoes of OVS are under hormonal regulation, with considerable differences between different stages of the estrous cycle [13]. This finding is consistent with previous transcriptome and proteome studies in the oviduct during the estrous cycle [67,68,69,70].

**Figure 1:**
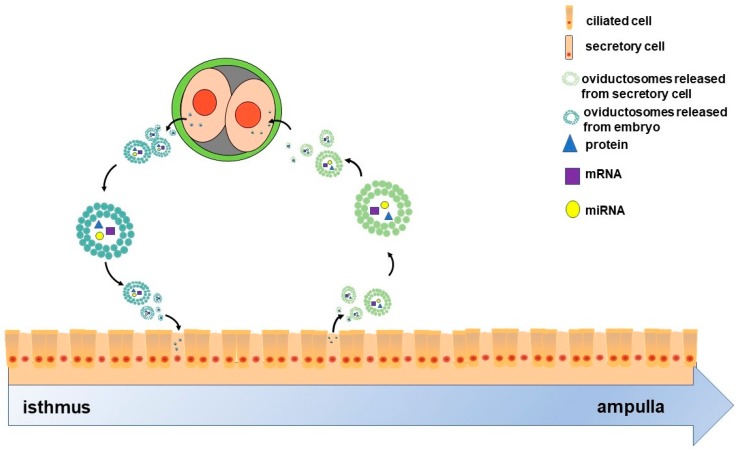
Scheme of the two-way communication between oviductal epithelial cells and embryos. The oviduct comprises ciliated and secretory cells. Oviductosomes (OVS)-containing proteins, mRNAs and miRNAs, are released from secretory cells, then internalized by two-cell embryos. These embryos also release OVS that are internalized by the epithelial cells. Ultimately, OVS as communicating mediators facilitate the cross-talk between oviductal epithelial cells and two-cell-stage embryos as two-way traffic.

On the other hand, the crosstalk between oviduct and embryo may not be unidirectional. It is well known that maternal recognition of pregnancy occurs in the uterus; however, oviduct also responded to embryo specifically. The oviductal epithelium can respond to signals from embryos by altering the composition of the fluid it secretes [71,72]. A number of pieces of evidence also indicated the impact of early embryo on the gene expression of oviduct epithelial cell. For instance, in mice, the presence of early embryos upregulated the expression of genes such as *Tβ4*, *Rpl41* and *MLC3nm* in oviduct [73]. In pigs, the presence of early embryos regulates the expression of *Ticam2*, an immune-related gene, in the oviduct epithelium, which depends on the embryo developmental stages [71]. Moreover, early embryos can also release EVs [74,75,76,77] that in turn might be components of OVS. Given this background, we hypothesized that oviduct may also respond to preimplantation embryos due to embryo-derived EVs. Overall, given their molecular cargoes, OVS as communicating mediators may facilitate cross-talk between the oviduct and preimplantation embryos as two-way traffic (Figure 1).

## 4. Oviductosomes: Possibilities for Optimizing In Vitro Culture Systems

Several key early embryo developmental events such as the first cleavage division and activation of the diploid embryonic genome, occur in the oviduct, and the embryos undergo epigenetic changes responsible for further development. However, embryos in in vitro conditions are inferior, as shown by lower blastocyst formation rates, altered inner cell mass/trophectoderm ratios and abnormal gene expression patterns [2,3,78]. Moreover, embryos produced under in vitro conditions have higher levels of DNA methylation than when growing in vivo [79]. It is worth mentioning that, despite considerable improvements in IVC systems designed to support early embryo development, such culture systems are still far from the normal physiological environment in the oviduct, where oviduct-derived soluble molecules, antioxidants, ciliary beating and oviduct-embryo crosstalk exist. Conventional IVC systems might compromise the developmental competence of preimplantation embryos in the absence of assistance from the oviduct [80]. Thus, the quality of embryos cocultured with oviductal epithelial cells is superior to that of embryos grown without coculture [5], so bovine oviductal epithelial cells can help overcome the developmental block at the 8- to 16-cell stage in IVC-produced cattle embryos [81]. In addition, culture systems using conditioned media also support the development of early embryos [82]. Thus, Lopera-Vásquez et al. [12] demonstrated that a medium conditioned with oviductal epithelial cells could improve blastocyst quality to the same extent as conventional coculture systems, and this positive effect could be reproduced by the sole addition of OVS isolated from such conditioned medium. Electron microscopy and nanoparticle tracking analysis also confirmed the presence of OVS in these types of conditioned media derived from the extended culture of oviductal epithelial cells monolayers [12].

Given this background, we hypothesized that current IVC conditions are suboptimal largely because OVS, which produce an ideal natural shuttle for carrying specific bioactive molecular cargo into preimplantation embryos, are absent. Up to now, supplementation with OVS during IVC has shown positive effects. For example, the supplementation of IVC media with OVS downregulates the expressions of the pregnancy recognition factor interferon-tau (*Ifnt)* and placenta-specific 8 (*Plac8*) genes that are linked to implantation [10]. Moreover, supplementation of IVC media with OVS might also alter the expressions of *Bax*, *Bcl2* and *Oct4* genes that associated with apoptosis and cell proliferation in embryos [12]. In addition, supplementation of IVC media with OVS also improved embryo development and cryosurvival [10,11]. Although suboptimal IVC conditions might lead to epigenetic effects on embryo developmental potential [83,84,85], supplementation of IVC media with OVS containing mRNAs linked to chromatin modification, such as histone methyltransferases, histone demethylases or DNA methyltransferase, will also partly revert the abnormal epigenetic modifications [13]. Thus, adding OVS into IVC media could deliver a cocktail of specific bioactive molecule into embryos and mimic the communication between oviductal epithelium and embryos and overcome the deficiencies in IVC systems. Furthermore, increasing our understanding of the content and function of OVS will provide great potential for optimizing IVC systems from a novel perspective.

Although this paper mainly discusses the role of OVS in optimizing in vitro culture systems, we shall also bear in mind that besides OVS, soluble molecules presented in oviductal fluid may also play vital roles in supporting embryo development. For example, oviduct-derived C3/C3b which was converted to IC3b, improves the development of embryos [51]. In addition, antioxidants in oviductal fluid may protect the embryos against reactive oxygen species in the microenvironment; large amounts of taurine and hypotaurine, which were synthesized and secreted by oviduct epithelial cells, have important antioxidant functions for embryos [86].

## 5. Oviductosomes: Potential Tools for Delivering Exogenous Nucleic Acids into Preimplantation Embryos

Due to the presence of the ZP barrier, zygotes and preimplantation embryos are remarkably resistant to the uptake of exogenous genetic material. It is also well known that because of a similar electrical charge to that of the cell membrane, nucleic acids are usually unable to pass such membranes [87,88,89]. In practice, the delivery of nucleic acids into preimplantation embryos mainly relies on microinjection, in vitro electroporation, viral transduction or liposomal transfection. Unfortunately, all these transgenic procedures have defects. For example, microinjection requires an expensive micromanipulator system and high-skill techniques. In addition, RNA interference (RNAi) experiments based on the microinjection of short interfering (si)RNA, can only be carried out on 1-cell zygotes [90,91,92], meaning that microinjection might not be suitable for RNAi experiments when the target genes need to be silenced at the 4-cell or 8-cell stages. The preparation and concentration of viral particles prior to infection are time-consuming and labor-intensive, so viral transduction based on viral vectors is inconvenient. In vitro electroporation has not made a significant advance in delivering nucleic acids into preimplantation embryos, because the ZP must be removed prior to electroporation; then, the ZP-free embryos tend to be trapped by the oviductal epithelium [93,94]. In addition, in vitro electroporation by itself can also cause damage [95]. Because the ZP acts as a barrier and blocks transfection, the efficiency of liposomal transfection is also low [96]. Thus, although preimplantation embryos devoid of the ZP could be transfected successfully [97], those transfected with liposomes also suffer similar shortcomings as embryos transfected by electroporation because of the absence of the ZP. The use of biocompatible nanomaterials with their large loading capacity, stability and highly specific affinity towards targeted cell populations [98,99] might prove to be a novel minimally invasive technique to deliver biomolecules into embryos, while the nonbiodegradable nature of nanomaterials limits their practical application [100]. Thus, a more effective delivery method for transfection with nucleic acids is highly desirable.

EVs, as nanosized biological vesicles, might provide a natural way to deliver functional nucleic acid molecules into recipient cells. This possibility has created much excitement among researchers seeking effective alternative delivery strategies. Using EVs as exogenous nucleic acid delivery vesicles has the following advantages. First, EVs are produced during natural cell cycles and are composed of endogenous cell membrane lipids and proteins, which allows them to more faithfully mimic the cell membrane than liposomes. Generally, liposomes, the synthetic vesicles, commonly interact with serum proteins or innate immune response molecules, and then may be destroyed by the immune system. However, it is well known that the surfaces of EVs have similar surface markers as cells, which prevents provocations of immune response [101]. Second, it is well known that free genetic material such as RNA molecules are fragile once in an extracellular environment. Several studies have shown that the lipid-rich membranes of EVs can serve to protect the internalized RNA molecules from degradation by extracellular RNases [102,103]. Third, EVs can also protect internalized RNA cargoes against environmental factors such as temperature changes. Ge et al. [104] have shown that EVs stored at –20 °C are able to retain their stored miRNAs unchanged for up to 5 years.

Two strategies have been used for loading EVs with nucleic acid molecules: during EVs biogenesis and after their isolation. Once the desired miRNA is introduced to cells, they can provide a continuous overexpression, and then EVs with an increased level of this miRNA, are released. This is because miRNAs are sorted to exosomes when the expression levels of endogenous mRNAs—the targets of miRNAs—are downregulated, and vice versa [105,106]. On the other hand, electroporation can also successfully introduce heterologous siRNAs into EVs. Briefly, to load EVs with heterologous siRNAs, isolated EVs are mixed with the siRNAs, then incubated on ice for 10 min and exposed to an electric field pulse [15]. Importantly, other studies have also shown that heterologous small RNAs, delivered by EVs, are functional in recipient cells [102,107,108].

Early embryos travel down the oviduct to reach the uterus, which provides a short time window but has huge consequences persisting into later stages of development. During this time, essential developmental events, such as first cleavage, zygotic gene activation (ZGA) and starting epigenetic reprogramming, occur in microenvironment of oviduct. More often, delivering exogenous nucleic acids into IVC-derived embryos needs to be completed prior to preimplantation stage. Nevertheless, embryos, cultured in IVC condition, bypass the embryo–oviduct interactions. OVS are the major mediators involved in communication between oviductal epithelium and preimplantation embryos. It is well known that the cell source of EVs may determine their biodistribution; that is to say, OVS can be targeted to preimplantation embryos. Therefore, the use of OVS might serve as the most physiologically appropriate method for delivering exogenous nucleic acids into preimplantation embryos, mimicking the natural mechanisms of molecular cargo trafficking. It is noteworthy that a novel exogenous nucleic acid delivery technology, called genome editing via oviductal nucleic acid delivery (GONAD), can directly deliver exogenous nucleic acids into preimplantation embryos in the oviduct in situ. Briefly, after surgical exposure of the oviduct, a solution of nucleic acids is instilled into the oviduct, then electric pulses are applied for electroporation into the embryos [109,110]. Unfortunately, besides the invasive surgery needed prior to electroporation, the GONAD technology also leads to poor fetal development because of the physical damage caused by electric pulses [109,110]. It is clear that oviductal epithelial cells release OVS into oviductal fluid that are involved in communication between the epithelium and preimplantation embryos. When combined with GONAD technology that can deliver exogenous nucleic acids into preimplantation embryos, we can assume that OVS might function as carriers of exogenous nucleic acids following electroporation and then deliver them into preimplantation embryos.

Currently, there are two methods available for obtaining OVS: from oviductal fluid aspirated in vivo, or from the secretion of oviductal epithelial cells subjected to IVC. Although OVS can be obtained from oviductal fluid, this source is limited, so oviductal epithelial cells subjected to IVC could provide an alternative source [10]. Cell polarity is defined as the asymmetric organization of cellular components, and this asymmetry can be associated with cell function, growth and survival [111]. The challenge now is to maintain cell polarity in IVC of oviductal epithelial cells as close as possible to normal. To preserve cell polarity and mimic the microenvironment of the oviduct, air–liquid-interphase (ALI) culture systems are needed for culturing in vivo-like primary oviductal epithelial cells [112,113]. Such ALI culture systems can secrete surrogate oviduct fluid and promote normal epithelial cell differentiation (e.g., polarization, columnar shape and ciliary activity), and functional epithelial tissues have been established in vitro [112,113]. Embryos cocultured with these oviductal epithelial cells in IVC systems can develop up to the blastocyst stage without the need for conventional embryo culture media [114]. Thus, using ALI culture systems, oviductal epithelial cells subjected to IVC can maintain their primary cell attributes and provide an alternative OVS source.

**Figure 2:**
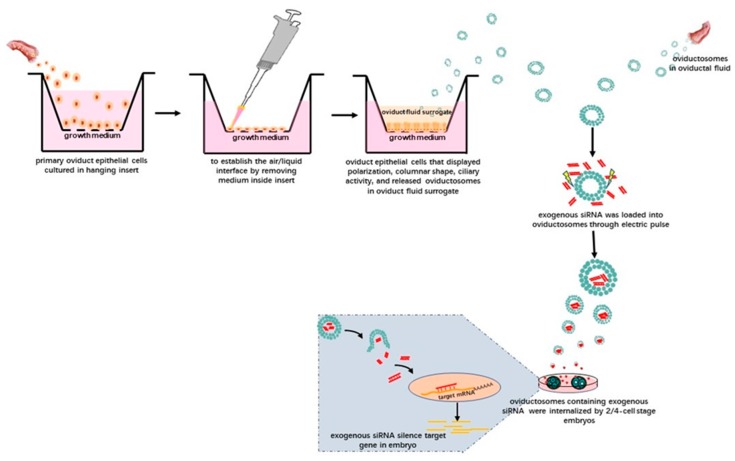
Schematic illustration showing the potential technology involved in delivering exogenous siRNAs into preimplantation embryos via OVS isolated from oviductal fluid. Alternatively, OVS could also be isolated from surrogate oviduct fluid secreted by oviductal epithelial cells cultured in an air–liquid-interphase (ALI) system and displaying normal morphological differentiation (e.g., polarization, columnar shape and ciliary activity). After isolating the OVS, exogenous siRNAs can be loaded into them by electroporation. After adding these OVS into the embryo in vitro culture (IVC) system, they can be internalized, eventually silencing target genes in the embryos.

Given this background, we suggest the following technical approaches. After isolating OVS from oviductal fluid or the secretions of oviductal epithelial cells subjected to IVC, exogenous nucleic acids—in theory—could be loaded into the purified OVS using electroporation. After adding these OVS into IVC media for preimplantation embryos and following the uptake of these OVS, the exogenous nucleic acids might be able to carry out their biological activities in preimplantation embryos. For instance, siRNAs could be transferred to preimplantation embryos via OVS (Figure 2). In this way, OVS, as physiologically normal intracellular carriers, might function as transmitters of exogenous nucleic acids. Moreover, only the OVS will be subjected to electroporation, leaving the preimplantation embryos free from the associated damage caused by electroporation.

It is noteworthy that cellular nanoporation biochips have now been developed, enabling the large-scale generation of functional mRNA-encapsulating exosomes. Yang et al. [115] cultured monolayers of source cells over the chip, which contained an array of nano-channels. These were subjected to transient electrical pulses that shuttled DNA plasmids from the buffer into the attached cells. This method yielded a more than 50-fold increase in secreted EVs and a more than 10^3^-fold increase in exosomal mRNA transcripts. We anticipate that the development of such novel biochip nanotechnology will provide exciting tools to load EVs and OVS with exogenous nucleic acids.

## 6. Challenges in Oviductosomes-Based Embryo Production

OVS provided promising tools for optimizing in vitro culture systems and delivering exogenous nucleic acids into preimplantation embryos, which only involves the production of transgenic livestock, not the clinical practice. However, there are still a number of challenges that need to be addressed. One major challenge is whether the sufficient number of OVS can be obtained. Besides oviductal fluid, oviductal epithelial cells subjected to IVC could provide an alternative OVS source, while the OVS yield per cell will impact the final production, and selecting donors should be critical. At the same time, because EVs play a vital role in pathological cellular processes (e.g., EVs are also secreted by a variety of cancer cells) [116], the health status of donors should also be given full attention in the process of selecting donors. The consistency of isolated OVS may be difficult to achieve as the OVS come from many different donors and possesses inherent variability, and then immortalization of excellent donor cells may maintain the consistency. Delivering *Myc* gene into excellent donor cells may allow these donor cells to obtain immortalization without altering the fundamental characteristics [117]. It is well known that there is still no consensus on markers that distinguish the origin of various EVs, and the absolute separation and definition of various EVs based on their size or biogenesis has not been established [118]. Therefore, OVS may also be composed of a heterogeneous population in terms of their biochemical composition, which prevents the use of OVS in clinic currently.

## 7. Conclusions

From an evolutionary perspective, OVS might act as conserved mediators of intercellular communication between the mammalian oviduct and preimplantation embryos. This reciprocal communication is not unidirectional, in that OVS are released from the oviductal epithelium and then taken up by preimplantation embryos, improving their development. At the same time, OVS derived from embryos can be taken up by the oviductal epithelium, eliciting maternal responses in terms of biological functions and pathways. Because of the potential bioactive cargoes contained in OVS, these provide new approaches for exploring the molecular mechanisms underlying intercellular communication during embryogenesis. The application of OVS might help to improve IVC systems for preimplantation embryos and provide exciting opportunities to develop novel methods for delivering exogenous genetic material into them, and in enhancing the production of transgenic animals. We anticipate that further investigations into the characteristics of OVS will open new horizons for research on maternal–embryo interactions and transgenesis in livestock.

## Abbreviations

EVs	Extracellular vesicles
ESCRT	Endosomal sorting complexes required for transport
OVS	Oviductosomes
IVC	In vitro culture
OVGP1	Oviduct-specific glycoprotein precursor 1
ZP	Zona pellucida
DNMT1	DNA methyltransferase
TPT1	Tumor protein translationally-controlled 1
ROS	Reactive oxygen species
RNAi	RNA interference
ZGA	Zygotic gene activation
GONAD	Genome editing via oviductal nucleic acid delivery
ALI	Air–liquid-interphase
